# Atrial fibrillation debut following first electroconvulsive therapy combined with venlafaxine: a case report and a literature review

**DOI:** 10.1192/j.eurpsy.2022.1434

**Published:** 2022-09-01

**Authors:** L. Ilzarbe, D. Ilzarbe, J. Gil, M. Valentí, O. De Juan, N. Arbelo, C. Llach, M. Bioque

**Affiliations:** 1 Hospital Clinic of Barcelona, Psychiatry And Psychology, Barcelona, Spain; 2 Department of Child and Adolescent Psychiatry and Psychology, Neuroscience Institute, Hospital Clinic Of Barcelona, University Of Barcelona, Idibaps, Cibersam, Barcelona, Spain; 3 Barcelona Bipolar Disorders Program, Neuroscience Institute, Hospital Clinic Of Barcelona, University Of Barcelona, Idibaps, Cibersam, Barcelona, Spain; 4 Hospital Clinic of Barcelona, University of Barcelona, IDIBAPS, CIBERSAM, Barcelona Bipolar Disorders Program, Neuroscience Institute, Barcelona, Spain; 5 Barcelona Clinic Schizophrenia Unit (BCSU), Neuroscience Institute, Hospital Clinic Of Barcelona, University Of Barcelona, Idibaps, Cibersambarcelona Clinic Schizophrenia Unit (bcsu), Neuroscience Institute, Barcelona, Spain

**Keywords:** Electroconvulsive therapy, Cardiovascular, Depression

## Abstract

**Introduction:**

Cardiovascular events (CVE) are infrequent adverse effects in patients receiving electroconvulsive therapy (ECT). Nonetheless, it constitutes a threat for patient’s life and may compromise continuing ECT.

**Objectives:**

To describe a case of acute-onset atrial fibrillation under combined therapy with ECT and venlafaxine.

**Methods:**

We present a 76-year-old man diagnosed of delusional disorder and without any previous CVE, who was hospitalized in our acute psychiatric unit by major depressive episode with psychotic symptoms resistant to pharmacological treatment (valproic-acid 100mg/d, haloperidol 6mg/d, venlafaxine 300mg/d). ECT was initiated presenting atrial fibrillation after first session of ECT, requiring amiodarone and anticoagulant treatment for stabilization. Second session of ECT was delayed for three-weeks, worsening the psychiatric symptoms. Haloperidol was discontinued initiating lurasidone with better cardiovascular profile.

**Results:**

CVE occur in 2% of the patients receiving ECT, being acute arrhythmia the most frequent one. Among them, few cases of atrial fibrillation (AF) under ECT have been reported. It has been hypothesised that initial vagal response followed by catecholamine surge secondary to ECT could facilitate the development of AF. In addition venlafaxine, an antidepressant drug, may also predispose to arrhythmia in high-risk individuals. High doses of venlafaxine (>300mg/d) combined with ECT have been related with an increment of CVE.

**Conclusions:**

Although clinically effective for the treatment of major depression disorder, combined therapy of ECT and venlafaxine could precipitate the start of a CVE in genetically susceptible individuals. Therefore, identify and clarify potential risk factors other than previous history of CVE is critical to reduce morbidity and mortality in these patients.

**Disclosure:**

No significant relationships.

